# Vitamin D receptor and vitamin D binding protein gene polymorphisms in patients with asthma: a pilot study

**DOI:** 10.1186/s12890-023-02531-3

**Published:** 2023-07-05

**Authors:** Daina Bastyte, Laura Tamasauskiene, Ieva Golubickaite, Rasa Ugenskiene, Brigita Sitkauskiene

**Affiliations:** 1 Department of Immunology and Allergology, Lithuanian University of Health Sciences, Kaunas, Lithuania; 2 Lab of Immunology, Department of Immunology and Allergology, Lithuanian University of Health Sciences, Kaunas, Lithuania; 3 Department of Genetics and Molecular Medicine, Lithuanian University of Health Sciences, Kaunas, Lithuania

**Keywords:** Asthma, Allergic asthma, Vitamin D, Vitamin D receptor, Vitamin D binding protein, Single nucleotide polymorphism

## Abstract

**Background:**

The effects of vitamin D are exerted by interaction with the vitamin D receptor (VDR) and vitamin D binding protein (VDBP). Polymorphisms in VDR or VDBP genes may affect vitamin D levels, influencing the pathogenesis of asthma and atopy. The aim of this study was to investigate the possible association of VDR and VDBP gene single-nucleotide polymorphisms (SNP), 25-hydroxyvitamin D (25(OH)D), blood eosinophils and total IgE level in subjects with asthma in comparison with healthy individuals.

**Methods:**

This case-control study enrolled 63 subjects with asthma (45 allergic and 18 non-allergic) and 32 healthy subjects were involved in the study. Sensitization of subjects to inhaled allergens was determined by a skin prick test, lung function was evaluated by spirometry. Blood eosinophil count was determined by standard methods. Serum 25(OH)D and total IgE levels were evaluated by ELISA. Polymorphisms in the VDR and VDBP genes on the 12q13.11 and 4q13.3 chromosomal region were analyzed using TaqMan SNP Genotyping Assay probes.

**Results:**

In asthma patients with vitamin D deficiency (< 20 ng/ml) the allele G of rs11168293 of VDR was more common than in those having insufficiency (20–30 ng/ml) of vitamin D (63% and 31%, p < 0.05). Moreover, asthmatic subject with rs11168293 G allele has significant higher blood eosinophil count compared to asthmatic without the rs11168293 G allele (8.5 ± 12.3% vs. 5.1 ± 1.5%, p < 0.05). Significantly higher IgE level was found in subjects with allergic asthma with the allele A of rs7041 on VDBP gene than in those without this allele (540 ± 110 and 240 ± 80 IU/ml, p < 0.05).

**Conclusions:**

The association of polymorphisms in VDBP and VDR gene, the rs11168293 G allele and the rs7041 A allele, with 25(OH)D, blood eosinophil and total IgE level in asthma, let us suggest that vitamin D, VDR and VDBP gene polymorphisms are important in pathogenesis of asthma despite its form in relation to atopy.

## Background

According to the literature approximately around 300 million people worldwide suffer from asthma [1]. Asthma is a chronic airway disease causing shortness of breath, chest tightness and cough [2]. In the majority of patients symptoms could be precipitated by exposure to allergens [3]. Allergen-induced (atopic) asthma is an inherited predisposition to produce IgE in response to exposure to environmental proteins [4]. Moreover, IgE is a marker of atopy and asthma phenotypes that are not related to specific IgE production are classified as non-allergic asthma [5–7]. Also, some asthma endotypes can have either allergic or non-allergic underpinnings and are typically characterized by some degree of eosinophilic airway inflammation [8].

It is known that vitamin D is subject of a great interest in research because of its potential to modulate the immune system from environmental and genetic aspects. Many studies have been made to understand how vitamin D impacts and regulates the immune system, but still not clear the underlying mechanisms of vitamin D impact and whether vitamin D deficiency contributes to asthma pathogenesis ^10^. Several studies have shown the relation of vitamin D and atopy or asthma [9–11]. It is known that vitamin D beneficially modulates diverse immunologic pathways in heterogeneous asthma endotypes, regulating the actions of immune cells to dampen excessive immune inflammatory responses [12, 13]. Moreover, vitamin D can affect the pathogenesis of asthma by suppressing the response of Th2 lymphocytes and reducing the production of IL-5, thereby decreasing the eosinophil counts and IgE levels. Vitamin D could be a potential regulator of blood eosinophils and serum IgE level. Several studies have shown that lower levels of vitamin D are associated with an increased blood eosinophil count in asthma [14, 15]. Other studies have noted that low vitamin D status is associated with higher IgE levels in atopic conditions [16–19]. On top of that, some studies assert that IgE levels and eosinophil counts can be higher in subjects with vitamin D deficiency or insufficiency than in those with sufficient levels of vitamin D [20–22], although there are controversial findings in the literature [23, 24].

Systemic vitamin D status is determined based on the serum 25-hydroxyvitamin D (25(OH)D) concentration. This metabolite has a long half-life (2 or 3 weeks) and the production of 25(OH)D in the liver is not tightly regulated. The function of vitamin D is applied via proper transport in circulation by vitamin D binding protein (VDBP also known as GC-globulin) [25]. VDBP binds to vitamin D metabolites and regulates the access of vitamin D to tissues and cells [25, 26]. Biologically active vitamin D metabolite and VDBP complex acts by binding to a vitamin D receptor (VDR) which heterodimerizes with the retinoid X receptor alpha (RXRα) [27, 28]. Vitamin D binding to VDR causes the heterodimer complex become activated which in turn recognizes vitamin D response elements (VDRE) resulting in gene transcription [28].

Previous studies let us suggest that polymorphisms in VDR and VDBP genes may be associated with changes in vitamin D levels and involved in pathological processes of asthma [29–31]. Some genetic studies have confirmed the relation between VDR gene variation and some diseases including asthma and atopic conditions [32, 33]. We hypothesized that polymorphisms in vitamin D pathway genes such as VDR and VDBP may affect vitamin D levels and influence higher IgE levels and eosinophil counts in asthma.

However, the exact mechanisms modulating the role of vitamin D in the pathogenesis of asthma are still a mystery. The aim of this study was to investigate a single nucleotide polymorphism (SNP) of VDR, VDBP gene in asthma cases and comparison with healthy individuals and to evaluate their relation to vitamin D in asthma.

## Methods

### Study design

This study was carried out at the Hospital of Lithuanian University of Health Sciences Kauno Klinikos included 63 adults (> 18 years) with mild to moderate persistent asthma who were diagnosed and classified according to Global Initiative for Asthma (GINA) recommendations and 32 healthy (non-sensitive) adults as a control group. The study was approved by the ethics committee, and informed consent was obtained from all the participants. Blood samples were collected during the period of September – April, from patients with asthma and healthy subjects who do not take vitamin D supplements, without any other conditions that may negatively influence study results. Sensitization to inhaled allergens was determined by skin prick test. Lung function of asthmatic patients was evaluated by spirometry using a CustovitM pneumotachometric spirometer (Custo Med, Ottobrunn, Germany). Blood samples from the subjects were divided into groups: based on sensitization to allergen (-s), samples were divided into allergic and non-allergic asthma groups.

### Sample collection and storage

Peripheral venous blood samples were collected from the subject into K3 EDTA tubes for investigation for the separation of DNA and RNA for genomic and expression studies and for eosinophil count. Samples for total IgE and 25(OH)D assay were drawn into serum tubes. The blood specimen was given a personal identifier number that was used to link and maintain the biological information derived. Serum tubes were centrifuged at 3500 rpm for 10 min, serum was separated and frozen at − 70 °C for further analysis.

### DNA extraction and SNP genotyping

DNA was isolated using the QIAamp DNA blood mini kit (Qiagen, Hilden, Germany) according to manufacturer’s instructions. Eight gene polymorphisms in the VDR (rs7975232, rs1544410, rs731236, rs3847987, rs2228570, rs11168293), and VDBP (rs4588, rs7041) genes in chromosome regions 12q13.11 and 4q13.3 were analyzed using TaqMan SNP Genotyping Assays probes (Thermo Fisher Scientific, CA, USA), respectively, according to the manufacturer’s protocol.

### Evaluation of vitamin D, blood eosinophils and total IgE

Measurements of serum 25(OH)D were performed by the enzyme-linked immunosorbent assay ELISA using DIAsource 25OH vitamin D Total ELISA kit (Louvain-la Neuve, Belgium). The analysis kit detection limit was defined as the apparent concentration two standard deviations below the average OD at zero binding was 1,5 ng/mL. Analysis of data was performed according to vitamin D content: deficiency < 20 ng/ml (< 50 nmol/L), insufficiency 20–30 ng / ml (50–75 nmol/L), normal amount 30–50 ng/ml (75–125 nmol/L) [34–37].

Blood eosinophils evaluation was performed with an automated hematology analyzer (Sysmex, Kobe, Japan).

Measurements of total IgE level in serum were performed by ELISA using commercial test kit (ELISA, Bio-Clin-Inc., St. Louis, USA).

### Sample size and statistical analysis

Sample size was estimated according to the data of asthma incidences in the Lithuanian population [38], using standartized sample size calculation formula (minimal number of required study subjects is 30).

All statistical analyses were performed on IBM SPSS Statistics, version 29 (IBM Corp., Armonk, NY, USA) and Microsoft Excel (Microsoft, Redmond, WA, USA). Methods of statistical analysis were selected after performance of Kolmogorov–Smirnov test. The significance of the differences between the group of patients and the asthma control group was assessed using the Student’s t-test (means, Gaussian populations), Mann–Whitney U test (medians, non-Gaussian populations) or Kruskal-Wallis test (medians, non-Gaussian populations), and Fisher’s exact test (proportions) were used. P values less than 0.05 were considered as significant in all cases. The odds ratios (OR) were calculated from allelic frequency with 95% confidence interval (95% CI) for the polymorphism of the VDR gene. Power analysis of the study was 60%. Evaluating the association between VDR, VDBP gene SNPs, eosinophil and total IgE level in subject with asthma study power was 80%.

## Results

### Demography state of studied subject

The demographic characteristics of the study groups are shown in Table 1. Studied groups did not differ significantly according to the study subject age. Subjects with allergic asthma had significantly higher total IgE levels than subjects with non-allergic asthma. There were no significant differences of blood eosinophils and lung function parameters between subjects with asthma despite their allergic status. In this study samples were divided into groups based allergen sensitization status (allergic, non-allergic asthma), vitamin D content (deficiency < 20 ng/ml, insufficiency 20–30 ng/ml, normal amount 30–50 ng/ml) and assessed the type of VDR gene polymorphisms and VDBP gene polymorphisms in asthma subjects and control group.

**Table 1:** Demographic characteristics of the study groups

	Subjects with asthma (n = 63)		
	**Allergic asthma (n = 45)**	**Non-allergic asthma (n = 18)**	**Control group (n = 32)**
**Male/female, N**	16/29	1/17	13/19
**Age, years**	41 ± 2	51 ± 2	37 ± 2
**Total IgE (kU/L)**	420 ± 70*	70 ± 20	N/A
**Blood eosinophils** **(% of leukocytes)**	5.9 ± 0.6	4.2 ± 0.7	N/A
**Lung function:**			
**FEV1 (l)**	3.2 ± 0.1	2.4 ± 0.2	N/A
**FEV1 (% from predicted value)**	96.2 ± 2.5	90.1 ± 6.7	N/A
**FVC (l)**	4.1 ± 0.2	3.2 ± 0.2	N/A
**FVC (% from predicted value)**	105.4 ± 2.5	104.0 ± 6.9	N/A
**FEV1/FVC (ratio)**	0.9 ± 0.3	0.8 ± 0.52	N/A

Values are presented as mean ± SEM unless otherwise indicated

*p < 0.05 compared between allergic and non-allergic asthma groups

FEV1 – forced expiratory volume in one second

FVC – forced vital capacity

### VDR and VDBP SNP genotyping in asthma group and healthy subjects

In the study were analyzed six SNPs in the VDR (rs7975232, rs1544410, rs731236, rs3847987, rs2228570, rs11168293), and two SNPs in VDBP (rs4588, rs7041) genes in chromosome regions 12q13.11 and 4q13.3 (Table 2). The study revealed that VDBP gene rs4588 GT genotype was less common and TT more common in subjects with non-allergic asthma than in the control group (24 vs. 53 and 24 vs. 3%; respectively, p < 0.05). There was no statistically significant difference in VDR gene polymorphisms between study groups. The genotype and allele frequencies of the analyzed VDR and VDBP SNPs in subjects with allergic asthma, non-allergic asthma and control group are presented in Table 2.

**Table 2:** VDR and VDBP polymorphisms (%) in studied groups

VDR gene variant	Genotype	Allergic asthma (n = 45)	Non-allergic asthma (n = 18)	Control group (n = 32)
rs7975232	AA AC CC	31 42 27	6 50 44	31 5 28
rs1544410	CC TC TT	42 49 9	65 35 0	37 50 13
rs731236	AA AG GG	44 49 7	67 33 0	36 50 14
rs3847987	CC CA AA	78 13 9	78 22 0	8 16 3
rs2228570	GG AG AA	27 56 18	18 53 29	28 53 19
rs11168293	GG GT TT	49 31 20	22 50 28	38 44 19
**VDBP gene variant**	**Genotype, major allele**	**Allergic asthma** **(n = 45)**	**Non-allergic asthma** **(n = 18)**	**Control group** **(n = 32)**
rs4588	GG GT TT	52 36 12	53 24* 24*	43 53 3
rs7041	AA AC CC	18 48 35	25 31 49	16 52 32

*p < 0.05 compared to control group

### Analysis of serum 25(OH)D level

Serum 25(OH)D level was statistically significantly lower in allergic and non-allergic asthma patients than in healthy subjects (14.0 ± 0.8 and 15.4 ± 1.0 vs. 22.6 ± 1.2 ng/ml; respectively, p < 0.01). There were no statistically significant differences of 25(OH)D levels between asthma groups despite allergic sensitization. According to 25(OH)D level, its deficiency was found more frequently in patients with allergic and non-allergic asthma compared to the control group (Table 3). Also no significant correlation was found between 25(OH)D level and blood eosinophil count, IgE levels or lung function parameters in any of the studied groups.

**Table 3:** Proportion of subject with asthma according to different serum 25(OH)D level

Serum 25(OH)D level	Allergic asthma ( n = 45)	Non-allergic asthma ( n = 18)	Control group ( n = 32)
Deficiency (< 20 ng/ml)	87 ( n = 39)*	83 ( n = 15)*	44 ( n = 14)
Insufficiency (20–30 ng/ml)	11 ( n = 5)*	17 ( n = 3)*	50 ( n = 16)
Normal (30–50 ng/ml)	2 ( n = 1)	0 ( n = 0)	6 ( n = 2)

*p < 0.01 compared to control group

### The association between VDR and VDBP gene polymorphisms and serum 25(OH)D level

The genotype and allele frequencies of the analyzed VDR and VDBP genes single SNPs in patients with asthma and control group between different serum 25(OH)D level groups are presented in Table 4. Rs11168293 G allele was more common in subjects with asthma who were found to be deficient in 25(OH)D than in those who were found to have insufficiency of this vitamin. No significant statistical differences were observed between different 25(OH)D level groups and VDR or VDBP gene variants in healthy subjects.

**Table 4:** The association between serum 25(OH)D level, genotype and allele frequencies (in %) of VDR and VDBP gene variants in asthmatic subject

VDR gene variant	Genotype, major allele	25(OH)D level asthma group		25(OH)D level control group	
		< 20 ng /ml n = 5	20–30 ng/ml n = 8	< 20 ng /ml n = 14	20–30 ng/ml n = 16
rs7975232	AA AC CC A allele	26 44 30 48	13 38 50 31	29 50 21	31 38 31
				54	50
rs1544410	CC TC TT C	49 43 8 71	50 50 0 75	31 46 23	40 53 7
				54	67
rs731236	AA AG GG A allele	52 42 6 71	50 50 0 75	31 46 23	39 54 8
				54	65
rs3847987	CC CA AA C allele	76 17 7 84	88 13 0 94	86 14 0	81 13 6
				93	88
rs2228570	GG AG AA G allele	36 50 14 61	38 50 13 63	36 50 14	38 50 13
				61	63
rs11168293	GG GT TT G allele	44 37 19 63*	13 38 50 31	43 36 21	38 44 19
				61	59
**VDBP gene variant**	**GENOTYPE, MAJOR ALLELE**	**25(OH)D LEVEL** **IN ASTHMA GROUP**		**25(OH)D LEVEL** **IN CONTROL GROUP**	
		< 20 ng/ml n = 54	20–30 ng/ml n = 8	< 20 ng/ml n = 14	20–30 ng/ml n = 16
rs4588	GG GT TT G allele	52 34 14 69	50 25 25 63	42	37
				58	56
				0	6
				71	66
rs7041	AA AC CC C allele	19 43 37 41	29 29 43 43	23 39 39	13 69 19
				42	47

*p < 0.05 compared vitamin D deficiency group with vitamin D insufficiency group in patients with asthma

### The association between VDR and VDBP gene polymorphisms and blood eosinophils in asthma

Study subjects with asthma and rs11168293 G allele had significantly higher blood eosinophil count (% of leukocytes) compared to asthmatic subjects without the rs11168293 G allele (Table 5). However, there was no statistically significant difference in genotype and eosinophil count between allergic and non-allergic asthma groups.

**Table 5:** The association between alleles of VDR, VDBP gene variants and blood eosinophils count (% of leukocytes) in subjects with asthma

VDR gene variant	Allele	blood eosinophil count (% of leukocytes)	p-Value
rs7975232	A C	7.8 ± 1.2 6.9 ± 1.6	0.22
rs1544410	C T	7.3 ± 1.3 7.7 ± 1.2	0.27
rs731236	A G	7.6 ± 1.3 7.7 ± 1.2	0.80
rs3847987	C A	7.7 ± 1.2 8.5 ± 3.	0.60
rs2228570	G A	7.6 ± 1.4 8.5 ± 1.7	0.81
rs11168293	G T	8.5 ± 1.3* 5.1 ± 1.5	< 0.05
**VDBP gene variant**	**Allele**	**blood eosinophil count (% of leukocytes)**	**p-Value**
rs4588	G T	7.7 ± 2.8 7.1 ± 2	0.58
rs7041	A C	7.2 ± 1.5 7.4 ± 1.5	0.45

Values are presented as mean ± SEM

* p < 0.05 compared genes different alleles

### The association between VDR and VDBP gene polymorphisms and total IgE level in asthma

Study results revealed that significantly higher IgE levels were found in subjects with allergic asthma with the allele A of rs7041 on VDBP gene than in those without this allele (Table 6). There was no statistically significant difference between VDR SNPs and studied groups.

**Table 6:** The association between alleles of VDR and VDBP gene variants and total IgE level in subject with asthma

VDR gene variant	Allele	Total IgE level, kU/l	p-Value
rs7975232	A C	430 ± 80 420 ± 90	0.71
rs1544410	C T	420 ± 70 470 ± 100	0.27
rs731236	A G	430 ± 90 500 ± 110	0.29
rs3847987	C A	450 ± 70 110 ± 50	0.08
rs2228570	G A	370 ± 70 370 ± 70	0.62
rs11168293	G T	420 ± 80 320 ± 80	0.20
**VDBP gene variant**	**Allele**	**Total IgE level, kU/l**	**p-Value**
rs4588	G T	400 ± 80 490 ± 120	0.19
rs7041	A C	540 ± 110* 240 ± 80	< 0.05*

Values are presented as mean ± SEM

* p < 0.05 compared genes different alleles

## Discussion

It was demonstrated that serum 25(OH)D level, blood eosinophil or total IgE level in asthma could be related to the single nucleotide polymorphisms in VDR or VDBP gene. We found statistically significant differences in the distribution of rs11168293 genotype variants in the VDR gene between asthma patients with vitamin D deficient (< 20 ng/ml) and asthmatic subjects with vitamin D insufficiency (20–30 ng/ml). Moreover, there was a significantly higher blood eosinophils count in asthmatic with rs11168293 G allele compared to asthma patients without the rs11168293 G allele. We also determined that rs7041 genotype of VDBP gene was related to total IgE level in asthma. There is a possibility that vitamin D and VDR may regulate immune markers that promote asthma.

In this study samples were divided into groups based allergen sensitization status (allergic, non-allergic asthma), vitamin D content (deficiency < 20 ng/ml, insufficiency 20–30 ng/ml, normal amount 30–50 ng/ml) and assessed the type of VDR gene polymorphisms (rs7975232, rs1544410, rs731236, rs3847987, rs2228570, rs11168293) and VDBP gene polymorphisms (rs7041, rs4588) in asthma subjects and control group. We observed that vitamin D deficiency was more frequent in asthmatics than in healthy subjects. This shows that vitamin D has anti-inflammatory effects and plays an important role in the pathogenesis of asthma. Moreover, several studies have found that low serum vitamin D levels are associated with increased exacerbations, increased airway inflammation, decreased lung function, and poor prognosis in asthmatic patients[39]. Experimental studies of animal models showed immunomodulation effects of vitamin D and VDR involving the Th2-driven inflammation in the lung [40]. In addition, are evidences that decreased vitamin D level induces secretion of IL-5, IL-6, and IL-8 which are important in pathogenesis of inflammation [41]. Also, vitamin D could downregulate the expression of MHC class II molecules and inhibit DC differentiation and maturation by preserving the immature phenotype of dendritic cells [42]. Most observational researchers have shown that vitamin D can be effective as an adjunctive treatment for asthma [43]. On the other hand, the findings of studies are controversial and do not unequivocally support a beneficial role of vitamin D in asthma or atopy [44, 45].

Studies revealed that some polymorphisms variant in VDR and VDBP gene can affect function of the receptor and contribute to circulating vitamin D concentration [46–48]. Hypothetically, low vitamin D levels in asthma and atopy may be associated with SNPs of the VDR and VDBP genes[45, 48–50]. For example, the study of Taiwanese and Mongolian populations demonstrated that Vitamin D plasma concentration lower than 40 ng/ml and VDR SNPs rs2228570 genotype, both contribute to increased susceptibility to bronchial asthma [9]. On top of that, studies revealed that VDR SNPs such as rs2228570 genotype have associations with lower levels of vitamin D [51]. In addition, meta-analysis study revealed that VDR rs7975232, rs1544410 and rs731236 gene polymorphisms may confer susceptibility to allergic diseases in certain populations [52]. Interesting, that asthma meta-analysis study showed positive findings for rs1544410 polymorphism in Caucasians, and for rs731236 polymorphism in Asians [52]. Although, several studies with asthma and atopy showed that some genotypes of VDR are associated with asthma, but not with vitamin D levels [53–55]. Our findings showed that G allele of variant rs11168293 on VDR gene was more common in subjects with asthma who were found to be deficient in vitamin D than in those with vitamin D insufficiency. We did not find statistically significant results between other genotypes of VDR and 25(OH)D levels. On the other hand, Galvao et al. study showed an association of several other polymorphisms in the VDR gene between vitamin D level in asthma and atopy, however in the study was not assessed the polymorphisms of rs11168293 on the VDR gene [33]. Unfortunately, there is a paucity of evidence regarding the relation of rs11168293 with vitamin D level or asthma. However, there is a possibility that different VDR gene variants may result in alteration of the biological effects produced by the binding of the receptor with vitamin D in the promoter regions of genes that respond to vitamin [56]. It could affect regulatory actions produced by immune systems cells and cause changes in immune markers of asthma. On the other hand, controversial results shows that potential associations of the VDR SNPs with asthma and atopy are unclear.

Study results revealed that asthmatic subjects with rs11168293 genotype G allele had significantly higher blood eosinophil count compared to asthmatic subjects without this allele. We did not find significant results between blood eosinophil count and 25(OH)D level, on the other hand, Filho et al. study showed that eosinophilic inflammation may be associated with lower vitamin D levels [21]. It is important that vitamin D via VDR prolongs eosinophil survival and increases the expression of membrane receptors that inhibit their apoptosis [57]. We hypothesize that such effects of vitamin D on eosinophils may be related to G allele of variant rs11168293 on the VDR gene and may lead to a lower need for new eosinophils. Unfortunately, the variant rs11168293 is poorly explored in vitamin D and eosinophilic inflammation contexts. The rs11168293 genotype of VDR should be further studied on a larger cohort to derive conclusive results.

In our study, we noted statistically significant differences in VDBP gene rs4588 GT and TT genotype in non-allergic asthma compared to the healthy controls. VDBP gene rs4588 GT genotype was less common and TT more common in subjects with non-allergic asthma than in the healthy subjects. On top of that, study of Kurdish population showed increased level of serum VDBP in asthmatic patients compared to the control group and revealed that variations in VDR and VDBP gene may impact on progression of asthma [50]. In addition, Shafi et al. study showed that CC genotype of rs4588 was linked to higher Vitamin D levels whereas the AA genotype was linked to lower levels of vitamin D in subjects with atopic dermatitis [48]. Persic et al. in the study with coronary artery disease patients revealed that VDBP gene rs4588 genotype GG was correlated with higher levels of vitamin D in the serum [58]. However, we found no significant difference between VDBP SNP’s and vitamin D levels. Also, we did not find a significant difference in VDBP gene polymorphi [50]. In addition, Iordanidou et al. also did not find an association between serum VDBP rs4588 genotypes and vitamin D level in asthma [59].

It was found that rs7041 genotype variant of VDBP gene was associated with IgE levels in asthma. In addition, Fawzy et al. study revealed that GG genotype and G allele of the rs7041 are potential risk factors for the development of asthma, while rs4588 AA genotype and A allele may play protective role [60]. Significantly higher IgE levels were found in subjects with allergic asthma with the rs7041 A allele than in those without it. On top of that, German Asthma Family Study found association between VDBP rs222040, rs7041 and total serum IgE [61]. VDBP is not only a carrier protein for vitamin D metabolites, but also involved in inflammation modulation [62, 63]. Several studies showed that VDBP is restricting the bioavailability of vitamin D to immune cells like monocytes, dendritic cells and therefore suppresses vitamin D receptor signaling [64, 65], it is important that VDBP also is associated with membrane-bound immunoglobulin on the surface of B-lymphocytes [66]. The rs7041 A allele of VDBP gene might be associated with allergen-induced inflammation.

We did not find a significant difference between VDR SNP and IgE level. Differently than eosinophils [57] IgE does not have a VDR but it is produced by B lymphocytes, vitamin D via VDR exerting an immunomodulatory effect to lymphocytes and directly or indirectly regulates the production of IgE in B lymphocytes [13, 67]. Hypothetically, that would explain why there is no relation between VDR polymorphisms and IgE.

Discrepancies between studies on VDR, VDBP gene SNP in atopy or asthma may be obtained due to different study populations with geographical and ethnical differences among the subjects involved in these studies. Moreover, the interaction between genes and environmental factors such as sun exposure, diet, or interactions of VDR gene with other genes involved in vitamin D metabolism in asthma and atopy may play an additional role, especially in complex diseases such as asthma. On top of that, inadequate limited sample sizes may also result in discrepancies in findings between the studies. This might be one of the reasons that our results are not in agreement with some other previous studies results.

To our knowledge, it is the first VDR and VDBP polymorphism study of asthma in the Lithuanian population, also there is limited available research data in The European population. Further studies are warranted to investigate the VDR and VDBP gene single nucleotide polymorphisms’ role in the immune system and to evaluate their relation with vitamin D level of asthma in different populations.

## Conclusions

Increased incidence of serum vitamin D deficiency in subjects with asthma, and association of the rs11168293 G allele on VDR gene with lower 25(OH)D level, higher blood eosinophil count (% of leukocytes) and the rs7041 A allele on VDBP gene with higher IgE level in atopic asthma, let us suggest that vitamin D and a number of its binding protein and receptor gene polymorphisms are important in pathogenesis of asthma.

## Data Availability

All data generated or analyzed during this study are included in this published article. The data presented in this study are available on request from the corresponding author.

## Abbreviations

ELISA: Enzyme-linked immunosorbent assay
FEV1: Forced expiratory volume in one second
FVC: Forced vital capacity
GINA: Global Initiative for Asthma
IgE: Total immunoglobulin E
IL: Interleukin
SEM: Standard error of mean
SNP: Single nucleotide polymorphisms
Th2: Type 2 helper T cells
VDBP: Vitamin D binding protein
VDR: Vitamin D receptor
