# Correction: Vitamin D receptor and vitamin D binding protein gene polymorphisms in patients with asthma: a pilot study

**DOI:** 10.1186/s12890-023-02579-1

**Published:** 2023-08-08

**Authors:** Daina Bastyte, Laura Tamasauskiene, Ieva Golubickaite, Rasa Ugenskiene, Brigita Sitkauskiene

**Affiliations:** 1 Department of Immunology and Allergology, Lithuanian University of Health Sciences, Kaunas, Lithuania; 2 Lab of Immunology, Department of Immunology and Allergology, Lithuanian University of Health Sciences, Kaunas, Lithuania; 3 Department of Genetics and Molecular Medicine, Lithuanian University of Health Sciences, Kaunas, Lithuania

**Correction: BMC Pulm Med 23, 245 (2023)**

**https://doi.org/10.1186/s12890-023-02531-3**

Following publication of the original article [1], the authors flagged that there was an error in the author list: the respective given and family names of the authors had been published the wrong way around. The author list has since been corrected in the published article and the corrected author list may be seen in this erratum. The authors thank you for reading this erratum.

## References

[1] Bastyte Daina, Tamasauskiene Laura, Golubickaite Ieva, Ugenskiene Rasa, Sitkauskiene Brigita (2023). Vitamin D receptor and vitamin D binding protein gene polymorphisms in patients with asthma: a pilot study. BMC Pulm Med.

